# Quantitative identification of senescent cells in aging and disease

**DOI:** 10.1111/acel.12592

**Published:** 2017-04-28

**Authors:** Anat Biran, Lior Zada, Paula Abou Karam, Ezra Vadai, Lior Roitman, Yossi Ovadya, Ziv Porat, Valery Krizhanovsky

**Affiliations:** ^1^ Department of Molecular Cell Biology Weizmann Institute of Science Rehovot 76100 Israel; ^2^ Flow Cytometry Unit Biological Services Department Weizmann Institute of Science 76100 Rehovot Israel

**Keywords:** aging, cancer, cellular senescence, flow cytometry, ImageStreamX, senescence‐associated beta‐galactosidase

## Abstract

Senescent cells are present in premalignant lesions and sites of tissue damage and accumulate in tissues with age. *In vivo* identification, quantification and characterization of senescent cells are challenging tasks that limit our understanding of the role of senescent cells in diseases and aging. Here, we present a new way to precisely quantify and identify senescent cells in tissues on a single‐cell basis. The method combines a senescence‐associated beta‐galactosidase assay with staining of molecular markers for cellular senescence and of cellular identity. By utilizing technology that combines flow cytometry with high‐content image analysis, we were able to quantify senescent cells in tumors, fibrotic tissues, and tissues of aged mice. Our approach also yielded the finding that senescent cells in tissues of aged mice are larger than nonsenescent cells. Thus, this method provides a basis for quantitative assessment of senescent cells and it offers proof of principle for combination of different markers of senescence. It paves the way for screening of senescent cells for identification of new senescence biomarkers, genes that bypass senescence or senolytic compounds that eliminate senescent cells, thus enabling a deeper understanding of the senescent state *in vivo*.

## Introduction

Cellular senescence is a multifaceted phenomenon that functions in tumor suppression, wound healing, embryonic development, biological aging, and development of pro‐inflammatory age‐related diseases (Campisi, 2013; Burton & Krizhanovsky, 2014; Munoz‐Espin & Serrano, 2014; Salama *et al*., 2014). Cellular senescence is characterized by an altered cell state that is activated in response to persistent DNA damage triggered by various stimuli such as telomere shortening, activated oncogenes, oxidative stress, and cell–cell fusion. Senescent cells are likely to have either beneficial or detrimental effects within tissues, depending on whether the cells are transiently or persistently present.

Our understanding of the role of cellular senescence in different biological contexts has been impeded in part by the difficulty of detecting their presence within tissues. Such detection is currently performed mainly by evaluation of senescence‐associated beta‐galactosidase (SA‐β‐gal) (Dimri *et al*., 1995; Debacq‐Chainiaux *et al*., 2009; Kuilman *et al*., 2010). However, SA‐β‐gal activity alone is not enough to allow us to conclude with confidence that cells are senescent, as positive staining can also occur in other biological contexts (Kuilman *et al*., 2010; Caldwell *et al*., 2011). Therefore, SA‐β‐gal staining is usually combined with staining for additional markers such as γH2AX—a marker for activation of DNA damage response. In addition, negative markers that should be absent in senescent cells can be used to exclude the cells that are not senescent. These markers indicate cell proliferation, like Ki67 or BrdU incorporation, or proteins ubiquitously present in the cell nuclei, but secreted from senescent cells and thus absent in their nucleus, like HGMB1 (Davalos *et al*., 2013). The SA‐β‐gal and each of the markers are usually evaluated separately in consecutive sections. This procedure is not only laborious and expensive but also does not allow multiple senescence biomarkers to be detected within the same cells, limiting the possibility of quantitative evaluation of senescent cells derived from tissues. Alternatively, SA‐β‐gal activity within cells can be quantified by flow cytometry using 5‐dodecanoylaminofluorescein di‐β‐D‐galactopyranoside as a substrate (Debacq‐Chainiaux *et al*., 2009). However, this method can be performed only on intact cells and therefore does not allow identification of intracellular markers in the same cells. Altogether, current methods do not allow detection and quantification of senescent cells in tissues based on combination of markers that is essential for their confident identification.

Conventional SA‐β‐gal staining fails to distinguish between different cell types that can be a source of senescent cells within complex tissues, limiting our understanding of the underlying biological phenomena. In an attempt to overcome the limitations of current methods for identification of senescent cells, we utilized ImageStreamX, an advanced imaging flow cytometer capable of producing multiple high‐resolution, fluorescent and bright‐field (BF) images of every cell directly in flow. Our approach combines the quantitative power of flow cytometry with high‐content image analysis. We modified the traditional SA‐β‐gal assay to meet the requirements of the ImageStreamX and performed the assay in a single‐cell suspension. Using this method, we identified and quantified senescent cells in tumors, fibrotic tissues, and normal tissues of young and aged mice.

## Results

### Utilizing ImageStreamX for the quantification of senescent cells

**Figure 1:**
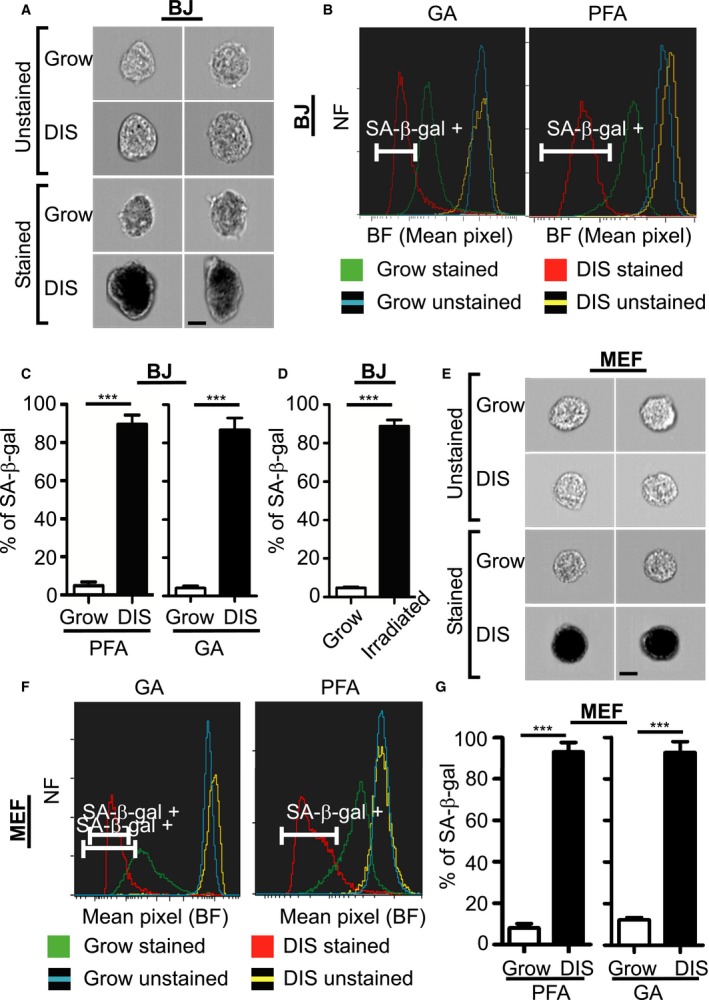
Identification and quantification of senescent cells *in vitro*. (A–G) Senescent and normally growing human BJ fibroblasts were stained for SA‐β‐gal or unstained and were analyzed by ImageStreamX. Bar, 10 μm. (A) Representative images of etoposide‐treated senescent BJ cells (DIS) and growing control cells after paraformaldehyde (PFA) fixation. (B) Bright‐field (BF) mean pixel intensity distribution of BJ cells after fixation with PFA or glutaraldehyde (GA). (C) Quantification of positive BJ cells as gated in (B). Data are presented as means ± SEM: for PFA,* n *≥* *6, performed in more than three independent experiments; for GA,* n *=* *4, performed in two independent experiments. ***P <* *0.001 (Student's *t*‐test). (D) Quantification of positive BJ cells that were irradiated to induce senescence, compared with growing cells; *n *=* *4, performed in two independent experiments. ***P <* *0.001. (E) Representative images of DIS mouse embryonic fibroblasts (MEFs) and control cells after PFA fixation. (F) BF mean pixel intensity distribution of MEFs after fixation with PFA or GA. (G) Quantification of positive primary MEFs as gated in (F). NF = Normalized frequencies. Values are means ± SEM; *n *=* *4, performed in two independent experiments; ***P <* *0.001(Student's *t*‐test).

To determine whether SA‐β‐gal staining can be detected and quantified using ImageStreamX technology, we subjected normal human BJ fibroblasts to DNA damage‐induced senescence by treating them with etoposide (DIS cells). Normal growing and DIS cells were fixed using either 4% paraformaldehyde (PFA) or 0.5% glutaraldehyde (GA) and were then stained for SA‐β‐gal and imaged by ImageStreamX. As a control, growing and DIS cells were fixed and analyzed without staining for SA‐β‐gal. Images of the cells revealed that the staining can be easily detected using bright‐field (BF) channel as cells positive for SA‐β‐gal appear in black (Figs 1A and S1A, Supporting information). To quantify the levels of SA‐β‐gal staining of each cell, we used the BF mean pixel feature, which exhibited superior separation ability compared with combinations of features (intensity, morphology, texture) with masks (Fig. 1B). The mean BF pixel intensity was calculated for each cell, such that SA‐β‐gal‐negative (bright) cells have high mean BF intensity, whereas SA‐β‐gal‐positive (black) cells have low mean BF intensity. Using either PFA or GA as the fixative agent, we observed a clear separation between DIS cells and growing‐untreated cells using ImageStreamX (Fig. 1B). To compare these results with a conventional microscopy, we subjected BJ cell suspensions to microscopic imaging. As expected, SA‐β‐gal‐positive cells were observed in DIS population (Fig. S1B, Supporting information). When fixed with PFA, SA‐β‐gal‐positive cells accounted for 89.6 ± 1.1% of DIS and for 4.8 ± 0.49% of growing BJ cells (*P *<* *0.001) (Fig. 1C). Similar results were obtained when we used GA as the fixative agent (SA‐β‐gal positive in 4.1 ± 0.71% of growing and 86.9 ± 2.3% of DIS cells, *P *<* *0.001) (Fig. 1C). The results are in line with those obtained when we quantified SA‐β‐gal‐positive cells by microscopy (Fig. S1C,D, Supporting information). Overall, these findings demonstrate that SA‐β‐gal staining can be detected and quantified using ImageStreamX. To determine whether this method of quantitation can be applied to cells induced to senesce by alternative stimuli, we used ionizing irradiation to induce BJ cells to senesce, and then stained them for SA‐β‐gal and analyzed them. ImageStreamX analysis revealed that 88.82 ± 3.2% of irradiated cells scored as SA‐β‐gal positive, compared with 4.7 ± 0.48% of growing cells used as controls (*P *<* *0.0001, Fig. 1D). To test the ability of this assay to detect senescent cells obtained from different species, we performed the assay on mouse embryonic fibroblasts (MEFs). These cells were subjected to treatment with etoposide to induce senescence (DIS), fixed with either PFA or GA, and stained for SA‐β‐gal. With either PFA or GA used as the fixative agent, clear differences were observed in SA‐β‐gal staining between DIS and growing cells (Fig. 1E,F). Quantification of SA‐β‐gal‐positive cells revealed that 93.0 ± 2.3% of DIS cells and 8.1 ± 1.0% of growing MEFs were scored as positive when fixed using PFA (*P *<* *0.0001) (Fig. 1G). Similar results were obtained with GA fixation (positive in 92.7 ± 3.7% of DIS cells and in 12.2 ± 0.75% of growing MEFs, *P *<* *0.0001) (Fig. 1G). Overall, these results demonstrate that ImageStreamX analysis can be used efficiently to identify and quantify SA‐β‐gal‐positive cells in both human and mouse cells.

### Combined staining of SA‐β‐gal with molecular markers for accurate quantification of senescent cells

**Figure 2:**
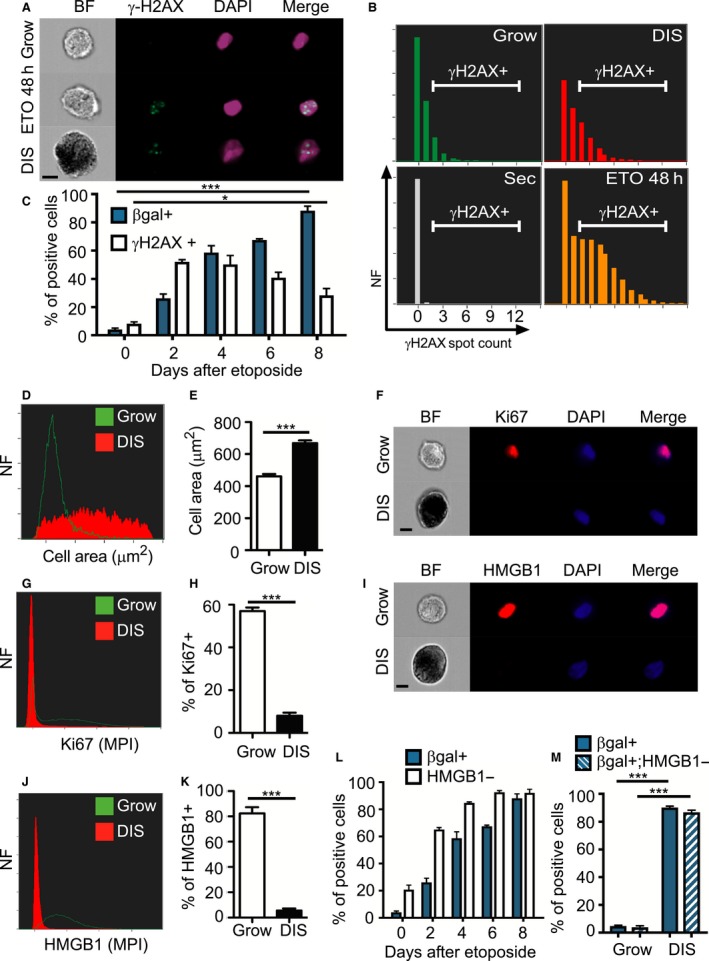
Identification of senescent cells via the combination of staining for SA‐β‐gal and molecular characteristics of senescence. (A–E) BJ cells treated with etoposide for 48 h, were harvested at the indicated times, stained for SA‐β‐gal, γH2AX (green), and DAPI (blue) and were analyzed by ImageStreamX. Control growing cells were considered time 0, cells immediately after the 48‐h treatment were considered day 2 and cells 8 days post etoposide treatment were considered as DIS. (A) Representative images of the cells stained as described above. Bar, 10 μm. (B) Distribution of numbers of γH2AX foci in these cells. DIS cells stained with the secondary antibodies only and DAPI served as a negative control (Sec). (C) Quantification of γH2AX‐positive and SA‐β‐gal‐positive BJ cells, as gated in (B), at different time points following initiation of etoposide treatment. (D) Representative histogram shows cell area distribution of DIS and growing BJ cells. (E) Quantification of cell area. (F–M) Growing and senescent BJ cells were stained for DAPI (blue) and Ki67 or HMGB1 (red) and were analyzed by ImageStreamX. Bar, 10 μm. (F) Representative images of the cells stained for Ki67. (G) Representative histogram presents max pixel intensity of Ki67 in DIS and growing BJ cells. (H) Quantification of Ki67‐positive BJ cells. (I) Representative images of cells stained for HMGB1. (J) Representative histogram showing mean pixel intensity of HMGB1 in DIS and growing BJ cells. (K) Quantification of HMGB1‐positive BJ cells. (L) Quantification of HMGB1‐negative and SA‐β‐gal‐positive BJ cells. *n* ≥ 3 (M) Quantification of the overlap between SA‐β‐gal staining and HMGB1‐negative staining. (A–C) Values are means ± SEM, *n* = 4 performed in two independent experiments. *P <* *0.05, ***P <* *0.001 (Student's *t*‐test). (E,H,K,M) Values are mean ± SEM, *n *≥* *5, and were replicated in two independent experiments. ***P <* *0.001 (Student's *t*‐test or two‐way ANOVA). NF = Normalized frequencies.

While staining for SA‐β‐gal is widely used to identify senescent cells, studies have shown that when this staining is combined with staining of molecular markers of senescence in the same cells, the resulting readout of the senescent state might be more reliable (Kuilman *et al*., 2010). The DNA damage response (DDR) is an important aspect of the senescence phenotype and its activation is frequently used as a molecular marker to identify senescent cells (Rodier *et al*., 2009; Kuilman *et al*., 2010; Campisi, 2013; Burton & Krizhanovsky, 2014; Munoz‐Espin & Serrano, 2014). Nuclear foci identified by staining for γH2AX (an early marker for DNA damage) are often used for this purpose. We therefore adjusted the method to combine staining for SA‐β‐gal with fluorescence staining for γH2AX. GA fixation resulted in good staining for SA‐β‐gal, but also yielded high autofluorescence compared to PFA fixation (Fig. S2A,B, Supporting information), making it unsuitable for use in combination with fluorescence staining. We therefore used PFA as our fixative agent. We also used channels yielding minimal differences in autofluorescence between senescent cells stained for SA‐β‐gal and unstained senescent cells (Fig. S2C, Supporting information), and between growing and senescent SA‐β‐gal‐positive cells (Fig. S2D, Supporting information). To follow up the accumulation of γH2AX foci over time, we treated BJ cells with etoposide for 48 h to induce DNA damage and analyzed the cells at different time points. We stained DIS and growing cells for SA‐β‐gal and γH2AX and stained with DAPI for nuclear DNA labeling. DIS cells subjected to staining with a secondary antibody only were used as a negative control for γH2AX staining. We were able to identify γH2AX foci localized within the nucleus, as well as SA‐β‐gal‐positive staining in DIS cells (Fig. 2A). Control growing cells were considered time 0, cell immediately after the 48‐h etoposide treatment were considered day 2 and DIS cells were considered 8 days post treatment Quantification of the γH2AX foci (Fig. 2B) disclosed that 7.8 ± 1.51% of growing cells, 28.3 ± 4.9% of DIS cells, and 52 ± 1.49% of the cells at day 2 were γH2AX positive (Fig. 2C). Although the cells at day 2 were highly positive for γH2AX, only 26.25 ± 2.95% of them were SA‐β‐gal positive, compared to 88.24 ± 3.7% of DIS cells (Fig. 2C). Importantly, among the γH2AX‐positive DIS cells, 91.3 ± 5.1% were SA‐β‐gal positive. Among growing and γH2AX‐positive day 2 cells, however, SA‐β‐gal‐positive cells comprised only 2.7 ± 3.2% and 22 ± 1.4%, respectively. The fraction of SA‐β‐gal‐positive cells was gradually increased from day 2 reaching its maximum at day 8. We further measured the cell area of growing cells and of DIS BJ cells. Clear differences were observed between them (Fig. 2D): the mean areas of DIS BJ cells and of growing cells were 668.9 ± 17.25 μm^2^ and 461 ± 14.8 μm^2^, respectively (Fig. 2E), implying that senescent cells are larger than growing cells.

Next, we stained DIS and growing BJ cells for SA‐β‐gal in combination with Ki67 or HMGB1 (Fig. 2F,I). In senescent cells, HMGB1 leaves the nucleus and relocates to the extracellular space (Davalos *et al*., 2013). Representative histograms revealed high levels of Ki67‐positive and HMGB1‐positive staining in the growing cells but hardly any detectable nuclear staining in the DIS cells (Fig. 2G,J). Further quantification revealed that 57 ± 1.7% of growing cells and 8 ± 1.4% of DIS cells were gated as positive to Ki67 (Fig. 2H). Dramatic differences were detected between senescent and growing cells stained for nuclear HMGB1, with 82.4 ± 4.9% positive growing cells comparing to 5.5 ± 1.6% positive DIS cells (Fig. 2K). No staining was detected in DIS or growing cells labeled only with secondary antibodies (Fig. S3A, Supporting information). To study the loss of nuclear HMGB1 during the establishment of senescent phenotype, we performed a time course experiment. BJ cells were treated with etoposide for 48 h; thereafter, cells were collected at the indicated times and analyzed for SA‐β‐gal staining and nuclear HMGB1 staining. Loss of nuclear HMGB1 staining with time was observed along with increase in the percentage of SA‐β‐gal‐positive cells. For example, 2 days after etoposide administration 26.25 ± 2.95% of the cells were SA‐β‐gal positive and 65.2 ± 2.5% of the cells were nuclear HMGB1 negative (Fig. 2L). Thus, the reduction in HMGB1 nuclear expression is an earlier event than the induction of SA‐β‐gal activity.

As HMGB1 was abundant in growing cells and almost absent in DIS cells, we combined the SA‐β‐gal and the HMGB1 markers to better quantify senescent cells. DIS and growing cells were gated for SA‐β‐gal positive and HMGB1 negative. With this combination, 3.88 ± 1.0% of growing cells and 86.8 ± 1.4% of DIS cells were gated as positive, compared to 4.1 ± 0.94% and 88.24 ± 3.2%, respectively, when only the SA‐β‐gal‐positive gate was used (Figs 2M and S3B, Supporting information). Similar results were obtained when Ki67 was combined with SA‐β‐gal positive (Fig. S3C, Supporting information). Overall, the above findings show that staining for SA‐β‐gal can be combined with additional markers of cellular senescence to gain a more reliable and accurate evaluation of the senescent state than that obtained with SA‐β‐gal‐positive staining alone.

### Quantification of senescent cells *in vivo*

**Figure 3:**
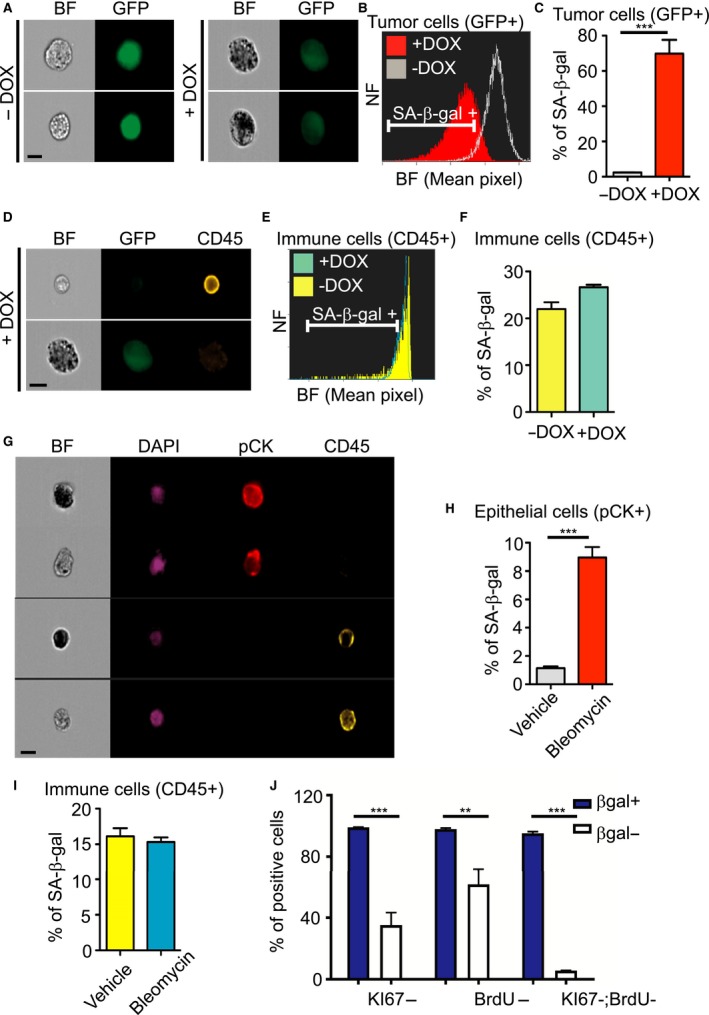
Identification and quantification of senescent cells *in vivo*. (A–F) MEF tumor cells expressing tetracycline transactivator protein tTA (‘tet‐off’), H‐ras^V12^, GFP, and TRE‐shp53 were injected subcutaneously into nude mice. Once tumors appeared, mice were treated with (+DOX) or without (−DOX) doxycycline. Tumors were extracted, dissociated, stained for SA‐β‐gal and CD45 (yellow), and analyzed by ImageStreamX. Bar, 10 μm. (A) Representative images of GFP‐positive tumor cells treated with or without DOX. (B) BF mean pixel intensity distribution of +DOX and −DOX tumor cells. (C) Quantification of positive tumor cells, as gated in (B). (D) Representative images of immune cells and tumor cells extracted from DOX‐treated mice. (E) BF mean pixel intensity distribution of immune cells extracted from DOX‐treated and untreated mice. (F) Quantification of positive immune cells, as gated in (E). (A–F) Data (*n *=* *5 mice) were replicated in two independent experiments. Values are mean ± SEM ***P <* *0.001 (Student's *t*‐test). NF = Normalized frequencies.(G–I) Lungs were extracted and dissociated from bleomycin‐treated and untreated mice. Cells were stained for SA‐β‐gal, CD45 (yellow), DAPI (blue), and Pan‐cytokeratin (PCK, red) and analyzed by ImageStreamX. (G) Representative images of the identified cells are shown. Bar, 10 μm. (H) Quantification of positive epithelial cells. (I) Quantification of positive immune cells. Data (*n *=* *4 mice) were replicated in two independent experiments. Values are means ± SEM, ***P <* *0.001 (Student's *t*‐test). (J) Quantification of Ki67‐ and BrdU‐negative cells within SA‐β‐gal‐positive and SA‐β‐gal‐negative lungs cells of bleomycin‐treated mice. *n* = 3, ***P <* *0.001, **P <* *0.01 (two‐way ANOVA).

Quantifying senescent cells in different tissues and organs is a major challenge. We therefore implemented this method to quantify senescent cells derived from mouse tissues. To address this in a comprehensive manner, we used two different *in vivo* systems. First, we used a well‐described system in which senescence is induced by reactivation of p53 (Dickins *et al*., 2005, 2007; Xue *et al*., 2007). We transduced MEFs with retroviruses expressing oncogenic *ras* (*H‐ras* ^*V12*^), the tetracycline transactivator protein tTA (‘tet‐off’), and tet‐responsive green fluorescent protein (GFP) together with shRNA targeting p53 (shp53). In agreement with previous reports (Dickins *et al*., 2005, 2007; Xue *et al*., 2007) in the absence of doxycycline (Dox), the expression of p53 protein was efficiently suppressed, whereas upon addition of Dox the expression of shp53 was shut off and p53 expression was restored, leading to cellular senescence (Fig. S4A,B, Supporting information). These transformed MEFs were injected subcutaneously into nude mice. As described previously (Dickins *et al*., 2005; Xue *et al*., 2007) in the absence of Dox, the mice rapidly developed tumors, and this was blocked upon Dox administration. Tumors were retrieved from Dox‐treated and untreated mice and were dissociated to single‐cell suspensions. Cells were analyzed for their viability, showing that 96.9% of untreated cells and 95% of Dox‐treated cells are viable following the dissociation (Fig. S4C, Supporting information). The cells were stained for SA‐β‐gal and CD45 to distinguish between tumor cells and immune cells. Tumor cells were gated according to their GFP‐fluorescence levels (Fig. S4D, Supporting information). Senescent tumor cells were identified in Dox‐treated mice (Fig. 3A,B), where 69.8 ± 7.8% of the tumor cells were scored as SA‐β‐gal positive compared with only 2.4 ± 0.07% SA‐β‐gal‐positive cells scored in untreated control tumor cells (Fig. 3C). Gating of immune cells according to their CD45‐positive staining and GFP‐negative fluorescence showed a clear separation between tumor and immune cells (Figs 3D, S4D,E, Supporting information). CD45‐positive cells extracted from Dox‐treated tumors showed a small elevation in their percentage of SA‐β‐gal‐positive cells relative to untreated mice tumors (Figs 3E,F and S4F, Supporting information). Overall, these results demonstrate both quantitative and cell‐type‐specific evaluation of SA‐β‐gal‐positive cells in different cell populations within complex tissues.

Secondly, to evaluate our method in an independent *in vivo* system, we induced pulmonary fibrosis in mice by treatment with bleomycin, shown to induce senescence in the lung (Aoshiba *et al*., 2013). Lungs from bleomycin‐treated and untreated mice were extracted and dissociated to produce single‐cell suspensions. The cells were stained for SA‐β‐gal, CD45 and pan‐cytokeratin (pCK) to identify immune cells and epithelial cells, respectively, and with DAPI to identify nuclei. Images acquired from cells derived from bleomycin‐treated mice indicated that immune and epithelial cells can be distinguished on the basis of their specific staining with CD45 and pCK, respectively, enabling us to quantify the SA‐β‐gal‐positive cells from each compartment (Fig. 3G). Among cells derived from bleomycin‐treated lungs, 8.96 ± 0.74% of the epithelial cells were scored as SA‐β‐gal positive compared with 1.14 ± 0.12% from control lungs (*P *<* *0.001) (Figs 3H and S5A, Supporting information). In the same mice, 15.3 ± 0.65% of the immune cells derived from bleomycin‐treated lungs were scored as SA‐β‐gal positive compared with 16.11 ± 1.14% from control lungs (Figs 3I and S5B, Supporting information). These results demonstrate efficient, quantitative, cell‐type‐specific identification of SA‐β‐gal‐positive cells in a whole organ. To understand the nature of SA‐β‐gal‐positive cells in this model, we combine the SA‐β‐gal staining with Ki67 staining and BrdU staining following BrdU injection into the mice. Our results demonstrate that 99.12 ± 0.41% of the SA‐β‐gal‐positive cells are also Ki67 negative and 98 ± 1.13% do not incorporate BrdU (Fig. 3J). Senescent cells were best separated from nonsenescent cells when we combined all these markers together (i.e. SA‐β‐gal; BrdU; Ki67). This analysis showed that 95.2 ± 1.9% of the SA‐β‐gal‐positive cells were negative for the proliferation markers, while only 5.57 ± 0.1% of the SA‐β‐gal negative were negative for the proliferation markers. These results indicate that SA‐β‐gal staining can be combined with Ki67 staining and BrdU incorporation analysis. Moreover, the results of the combined analysis further support nonproliferative nature of SA‐β‐gal‐positive cells identified in our assay.

### Quantification of senescent cells during aging

**Figure 4:**
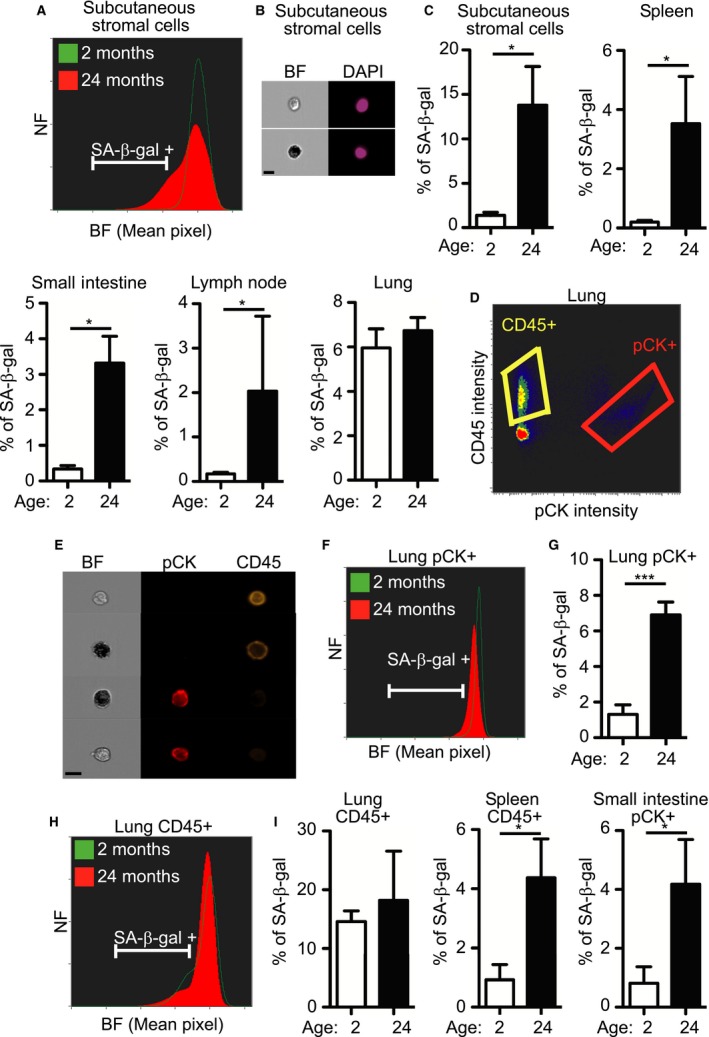
Identification and quantification of senescent cells in mouse tissues during aging. (A–I) Tissues extracted from 2‐ and 24‐month‐old mice were dissociated, stained for SA‐β‐gal and DAPI, and analyzed by ImageStreamX. Bar, 10 μm. (A) BF mean pixel intensity distribution of stromal cells derived from subcutaneous adipose tissue of 2‐ and 24‐month‐old mice. (B) Representative images of SA‐β‐gal‐positive and SA‐β‐gal‐negative subcutaneous adipose‐tissue‐derived stromal cells. (C) Quantification of SA‐β‐gal‐positive cells in the different tissues. (D) Tissues were stained for SA‐β‐gal, CD45, PCK, and DAPI. Immune cells were gated as CD45+ and epithelial cells as pCK+. (E) Representative images of CD45+ immune cells and PCK+ epithelial cells extracted from lungs. (F) BF mean pixel intensity distribution of PCK+ epithelial cells extracted from the lungs of 2‐ and 24‐month‐old mice. (G) Quantification of SA‐β‐gal‐positive PCK+ cells, as gated in (F). (H) BF mean pixel intensity distribution of CD45+ immune cells extracted from the lungs of 2‐ and 24‐month‐old mice. (I) Quantification of SA‐β‐gal‐positive lung CD45 +  cells as gated in (H). (A–I) NF = Normalized frequencies.Data (*n *=* *4 mice) were replicated in two independent experiments. Values are means ± SEM, *P <* *0.05 ***P <* *0.001 (Student's *t*‐test).

Senescent cells have been shown to accumulate in tissues with age (Dimri *et al*., 1995; Wang *et al*., 2009). To gain further insight into the role of senescent cells during aging, we implemented our method to identify and quantify senescent cells from various tissues in young (2‐month‐old) and old (24‐month‐old) mice. Lungs, lymph nodes, small intestines, spleen, and subcutaneous adipose‐tissue‐derived stromal cells were dissociated to single‐cell suspensions, stained for SA‐β‐gal and with DAPI, and analyzed by ImageStreamX for the percentage of SA‐β‐gal‐positive cells. Representative histograms of subcutaneous adipose‐tissue‐derived stromal cells from young and old mice show an increase in positive cells in the old animals (Fig. 4A). SA‐β‐gal‐positive and SA‐β‐gal‐negative cells can be clearly seen in images of these cells (Fig. 4B). Further quantification showed the percentage of senescent cells in the various young and old mouse tissues with age (respectively, 1.4 ± 0.35% and 13.8 ± 4.34% in subcutaneous adipose‐tissue‐derived stromal cells, 0.19 ± 0.06% and 3.53 ± 1.59% in spleen cells, 0.3 ± 0.06% and 3.3 ± 0.76% in small intestinal cells, 0.16 ± 0.03% and 1.48% ±1.1% in lymph node cells, and 5.9 ± 0.86% and 6.7 ± 0.6% in lung cells).

To gain additional insights, we stained lung cells with pCK (for epithelial cells) and CD45 (for immune cells) antibodies and gated the cells into different subpopulations (Fig. 4D,E). Representative histograms of pCK+ or CD45 +  cells extracted from the lungs of young and old mice demonstrated differences in SA‐β‐gal staining in the two cell populations (Fig. 4F,H). Quantification of SA‐β‐gal‐positive cells in the pCK+ population revealed SA‐β‐gal‐positive cells in 1.3 ± 0.53% of young and 6.9 ± 0.73% of old mouse lung tissues (*P *<* *0.001) (Fig. 4G). Quantification of SA‐β‐gal‐positive cells in CD45 +  populations showed no significant differences between young and old mice. Importantly, significant differences between young and old mice were not observed in our analyses of total cells from the lungs, but only when the cells were separated into the different cell‐type populations. Analysis of each population by itself might therefore add some valuable insights and lead to identification of cell populations where percent of senescent cells is different.

**Figure 5:**
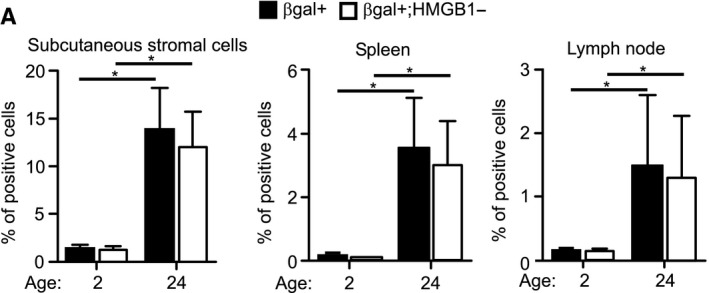
Lack of HMGB1 staining in SA‐β‐gal‐positive cells in mouse tissues. (A) Tissues extracted from 2‐ and 24‐month‐old mice were dissociated, stained for SA‐β‐gal, HMGB1, and DAPI, and analyzed by ImageStreamX. Quantification of the overlap between SA‐β‐gal‐positive staining and HMGB1‐negative staining in the different tissues. Data (*n *=* *3 mice) were replicated in two independent experiments. Values are mean ± SEM; *P <* *0.05 (two‐way ANOVA).

**Figure 6:**
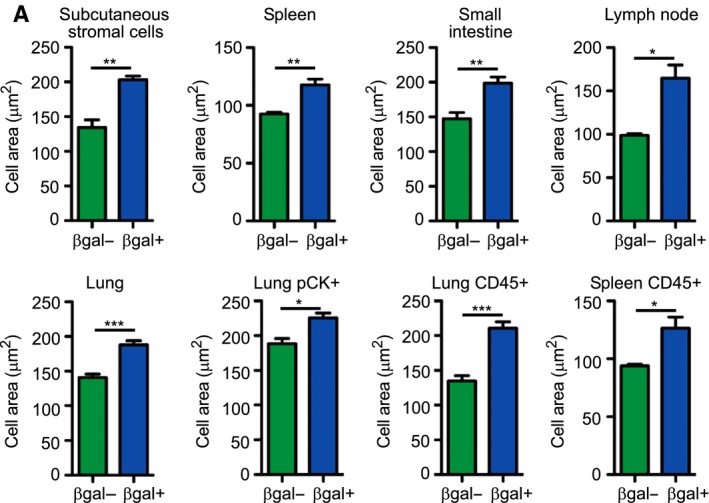
Increase in size of SA‐β‐gal‐positive cells in 24‐month‐old mice. (A) Cells from the different tissues were gated as SA‐β‐gal positive or SA‐β‐gal negative and their area was analyzed by ImageStreamX on BF images. Data (*n *=* *4 mice) were replicated in two independent experiments. Values are mean ± SEM; *P <* *0.05, **P <* *0.01, ***P <* *0.001 (Student's *t*‐test).

To gain a reliable and accurate evaluation of the senescent state during aging, we combined staining for SA‐β‐gal with staining for HMGB1. Subcutaneous adipose‐tissue‐derived stromal cells and cells from spleen and lymph nodes were stained for SA‐β‐gal and HMGB1 and gated for SA‐β‐gal‐positive, HMGB1‐negative population. A small reduction in the percentage of cells gated as positive for both stains was observed when compared with gating by SA‐β‐gal only (Fig. 5). Further studies are needed to investigate the correlation between staining and the lack of HMGB1 staining *in vivo*; nevertheless, this experiment demonstrated that the method can be used to combine staining with additional markers within the same cell. We took further advantage of the ability of the method to gate SA‐β‐gal‐positive and SA‐β‐gal‐negative cells to determine the sizes of these two cell populations (Fig. S6, Supporting information). Analysis of cell size in the different tissues of old mice showed an increase in the SA‐β‐gal‐positive population in all tissues tested (Fig. 6). These results demonstrated that senescent cells *in vivo* are larger than normal cells.

In conclusion, we showed here, both *in vitro* and *in vivo*, that senescent cells can be quantified when conventional SA‐β‐gal staining is combined with ImageStreamX technology. The method makes it possible to evaluate senescent cells quantitatively in many tissues during damage, aging, and disease. Furthermore, it can be combined with additional markers for cellular senescence to provide a reliable and accurate evaluation of presence of senescent cells in tissues.

## Discussion

For the past 20 years, the SA‐β‐gal assay has been viewed as the gold standard for identification of senescent cells, both in cell culture and in tissue samples (Dimri *et al*., 1995; Debacq‐Chainiaux *et al*., 2009; Kuilman *et al*., 2010; Caldwell *et al*., 2011; Campisi, 2013; Munoz‐Espin & Serrano, 2014). Within tissues, detection of senescent cells is often limited to observation of SA‐β‐gal activity within frozen sections. Furthermore, current SA‐β‐gal protocols do not allow for precise quantification of senescent cells in tissues, or the combined use of other molecular cell senescence markers within the same tissue sample, or identification of the specific cell types that have undergone senescence. These additional analytical approaches would greatly enhance our ability to understand the biology of cell senescence *in vivo*. They will also provide a platform for combining several markers for more reliable identification of senescent cells, or any other cell type that requires combination of markers for their identification.

We have shown here that senescent cells from various tissues can be quantified by simultaneous analysis of conventional SA‐β‐gal activity in BF and the use of additional senescence biomarkers via fluorescence labeling. Using this novel approach, we successfully quantified senescent cells in mouse tumors and fibrotic lungs. Importantly, our ImageStreamX quantification is consistent with previously published microscopic quantification demonstrating that about 8% of the lung pan‐cytokeratin‐positive cells are SA‐β‐gal 14 days after bleomycin treatment (Aoshiba *et al*., 2013). We also carried out the first quantitative examination of senescent cells present in different tissues of aged mice. Our findings clearly demonstrate the superiority of the method we developed for analysis of senescent cells over the traditional older techniques. Using this method, we were able to provide a quantitative cell‐type‐specific evaluation of presence of senescent cells in tissues.

Several studies have shown that p16^Ink4a^‐positive senescent cells accumulate with age in multiple tissues (Janzen *et al*., 2006; Krishnamurthy *et al*., 2006; Molofsky *et al*., 2006) and that their clearance successfully delays aging and age‐related pathologies in mouse models (Baker *et al*., 2011, 2016). The clearance of p16^Ink4a^‐positive cells was associated with reduction in SA‐β‐gal activity detected *in situ* by electron microscopy. Given the limitation of SA‐β‐gal staining on its own (Severino *et al*., 2000; Lawless *et al*., 2010), its combined analysis with additional senescent markers such as p16^Ink4a^, Ki67, and BrdU analysis would have provided additional robustness and insight. Moreover, recent publications have demonstrated the effectiveness of pharmacological agents in specifically inducing cell death in senescent cells *in vivo* (Chang *et al*., 2016; Yosef *et al*., 2016). Our method could be utilized to assess robustly and quickly the effectiveness of such compounds that specifically eliminate senescent cells from tissues.

In this study, we evaluated several biomarkers of cellular senescence and found a significant correlation between SA‐β‐gal staining and the lack of nuclear HMGB1 staining *in vitro*. This combination might allow more reliable identification of senescent cells, compared to SA‐β‐gal assay alone. Therefore, it provides significant advantage over existing techniques, including the use of fluorescent β‐gal substrate, which does not allow combination staining with any intracellular molecular markers. Accordingly, it seems possible to take advantage of this method to screen for new senescence biomarkers that correlate with SA‐β‐gal activity *in vivo*, and would consequently open the way to a deeper understanding of the senescent state *in vivo*. Furthermore, the use of senescence biomarkers will potentially yield greater biological insight by allowing protein localization and colocalization to be monitored and compared between senescent and nonsenescent cells.

Through its use of cell‐type‐specific biomarkers, our protocol can successfully determine which cell types undergo cellular senescence and which do not. Importantly, in the experiments with mice of different age, SA‐β‐gal staining was performed for 12 h in all tissues to ensure consistency. We suggest that in future studies SA‐β‐gal staining time has to be calibrated for each tissue and in some circumstances even different cell population, to achieve the most accurate results. Moreover, staining of the cells for live‐dead markers immediately following tissue dissociation will allow quantification of SA‐β‐gal‐positive cells specifically from the live cell population. This is particularly pertinent since the dissociation of cells from tissues might result in a certain amount of cell death. We showed that about 96% of the cells are viable following tumor dissociation, but this percentage can diverse greatly depending on the tissue examined.

Finally, using the ImageStreamX protocol, we successfully evaluated the sizes of senescent cells from tissues. Although an increase in the size of senescent cells is observed in tissue culture, there are hardly any reports of changes in senescent cell size *in vivo*. Owing to the ability of our method to gate and analyze senescent and normal cells in parallel, this question can be easily addressed and senescent cells size can accordingly be measured in different experimental setups *in vivo*.

In summary, our protocol provides a relatively quick, cost‐efficient, quantitative solution for detecting the presence of senescent cells and their cellular origins within tissues, thereby providing a better understanding of the role of senescent cells in physiological and pathological conditions. Senescent cells can play important roles in tumor suppression, tissue damage, aging, and embryonic development (Burton & Krizhanovsky, 2014; Munoz‐Espin & Serrano, 2014). They interact with the immune system to regulate their presence and modify the microenvironment (Xue *et al*., 2007; Krizhanovsky *et al*., 2008; Lujambio *et al*., 2013; Biran *et al*., 2015; Sagiv *et al*., 2016). Given the broad spectrum of biological contexts within which senescent cells function, it is anticipated that the wealth of information gained by quantitative analysis of senescent cells based on multiple characteristics will greatly enhance our understanding of senescence biology.

## Experimental procedures

### Cell culture

Cells were cultured in Dulbecco's modified Eagle medium (DMEM) supplemented with 10% fetal bovine serum (FBS) and 1% penicillin–streptomycin at 37 °C and 5% CO_2_ and were then passaged using trypsin. To induce senescence, we treated BJ cells and MEFs for 48 h with 20 and 50 μm etoposide, respectively, or irradiated them with 8 Gy and cultured for an additional 6–7 days. To induce quiescence, BJ cells were cultured for 3 days after full confluence was reached. To induce acute DNA damage, we treated BJ cells with 50 μm etoposide for 48 h.

### SA‐β‐galactosidase staining

Cells were collected in 15‐mL tubes, rinsed in 1 mL phosphate‐buffered saline (PBS) and fixed by adding 1 mL 8% paraformaldehyde (PFA) while vortexing (4% final PFA concentration) for 5 min or 1 mL 1% glutaraldehyde (GA, for 0.5% final concentration; both Sigma‐Aldrich, Rehovot, Israel) for 15 min at room temperature. Cells were washed once with PBS and then with PBS/1 mm MgCl_2_, pH 6.0 for human cells or pH 5.5 for mouse cells. Cells were resuspended in 5 mL of freshly prepared 5‐bromo‐4‐chloro‐3‐indolyl‐β‐D‐galactopyranoside (X‐Gal) staining solution (Inalco, San Luis Obispo, CA, USA) and incubated horizontally at 37 °C, sealed, and protected from light for 4 h (GA‐fixed) or 8–12 h (PFA‐fixed). Cells were then washed twice with 15 mL PBS and analyzed by ImageStreamX or stained with specific antibodies.

### X‐Gal staining solution

5‐Bromo‐4‐chloro‐3‐indolyl‐β‐D‐galactopyranoside (X‐Gal; Inalco) was dissolved in dimethylformamide (Sigma‐Aldrich, Rehovot, Israel) to a stock concentration of 100 mM. Potassium ferricyanide (K_3_Fe(CN)_6_ powder) (Merck, Darmstadt, Germany) and K_4_Fe(CN)_6_ 3H_2_O (Sigma‐Aldrich) were dissolved in double‐distilled water to a stock concentration of 200 mm. Staining solution was prepared by mixing PBS/MgCl_2_ with 5 mm K_3_Fe(CN)_6_, 5 mm K_4_Fe(CN)_6_ 3H_2_O and 2.5 mm X‐Gal.

### Immunofluorescence staining

Following staining for SA‐β‐gal, the cells were fixed with fixation buffer (eBioscience, San Diego, CA 00‐5223‐56) for 30 min at 4 °C, washed twice with permeabilization buffer (eBioscience), and incubated overnight with anti‐γH2AX primary antibodies, Ki67 (both Cell Signaling, Danvers, MA, USA), BrdU (Bio‐Rad, Hercules, CA, USA), or HMGB1 (Abcam, Cambridge, UK). Cells were washed twice and were then incubated for 45 min with secondary antibodies (Jackson ImmunoResearch Baltimore Pike, West Grove, PA, USA), followed by two additional washings and staining with DAPI for 10 min. For samples from mice, cells were labeled for 1 h with APC‐ or BV605‐labeled CD45 (Biolegend, San Diego, CA, USA) and PE‐conjugated pan‐cytokeratin antibodies (Abcam). Cells were washed, incubated with DAPI for 10 min, and analyzed by ImageStreamX.

### ImageStreamX analysis

The ImageStreamX system is an advanced imaging flow cytometer, combining features of fluorescent microscopy and flow cytometry. Cells in suspension pass through the instrument in a single file where transmitted light, scattered light and emitted fluorescence are collected, at a rate of up to 5000 cells s^−1^. This is accompanied by a dedicated image analysis software (IDEAS), which allows advanced quantification of intensity, location, morphology, population statistics, and more, within tens of thousands cells per sample. It allows analysis of rare subpopulations in highly heterogenous samples and gives rise to novel applications that were difficult to achieve by either conventional flow cytometry or microscopy (Zuba‐Surma *et al*., 2007).

Cells were stained as described above and imaged by ImageStreamX mark II (Amnis, Part of EMD milipore ‐ Merck, Seattle, WA, USA). At least 3*10^4^ cells were collected from each sample. Images were analyzed using ideas 6.1 software (Amnis, Part of EMD milipore ‐ Merck, Seattle, WA). For *in vitro* assays, cells were gated for single cells using the area and aspect ratio features on the BF image. For *in vivo* assays, cells were gated for single cells using the area and intensity of DAPI. Cells were also gated for focused cells using the contrast and gradient RMS features. To quantify the intensity of SA‐β‐gal staining, we examined several combinations of features (texture, intensity, and morphology) and masks. The best separation between stained and control cells was obtained with the mean pixel feature (mean background‐subtracted pixels within the input mask) of the BF channel. Gating for positive cells was achieved by unstained cells as a reference and visual inspection of stained cells to verify the gating. To quantify γH2AX foci, we applied the spot‐count feature on a spot mask created for the γH2AX acquisition channel, thus separating bright spots from the background. Using the BF images, we measured the cell areas in SA‐β‐gal‐positive and SA‐β‐gal‐negative cells.

### *In vivo* study

All experiments were done with the approval of the Weizmann Institute Animal Care and Use Committee. A mouse model for bleomycin‐induced pulmonary fibrosis was generated as described previously (Aoshiba *et al*., 2013). Anesthetized mice were subjected to intratracheal administration of 40 μL of a PBS solution containing bleomycin hydrochloride (10 mg kg^−1^ body weight). For BrdU experiments, a single intraperitoneal injection of BrdU (100 mg kg^−1^) was given to mice, 8 h prior to lung isolation. At 14 days after bleomycin injection, the mice were killed and their lungs were removed, chopped, and dissociated to single‐cell suspensions by incubation for 1 h with RPMI medium supplemented with 0.5 mg mL^−1^ collagenase IV and 0.02 mg mL^−1^ DNase I at 37 °C. Cells were then filtered with a 100‐μm nylon filter mesh, washed twice with PBS, and stained for actosidase activity for 16 h. Cells were then stained for fluorescence markers.

Transformed MEFs expressing H‐ras^V12^, tTA, GFP, and TRE‐shp53 were injected subcutaneously into the rear flanks of nude mice (10^6^ cells per flank). Once tumors were visible, the mice were treated with 0.5 mg mL^−1^ doxycycline in 0.5% sucrose solution in lightproof bottles and refreshed every 4 days. Five days after termination of doxycycline treatment, tumor tissues were minced and digested in DMEM containing 1000 U mL^−1^ dispase for 40 min at 37 °C. Cells were filtered through a 100‐μm nylon mesh, washed twice with PBS, and stained for SA‐β‐gal activity for 16 h. For cells viability assay, cells were stained with Zombie UV dye (Biolegend) for 30 min at RT and then analyzed by ImageStreamX for the percentage of viable cells.

To quantify SA‐β‐G‐positive cells during aging, tissues were extracted (subcutaneous stromal cells, spleen, small intestine, mesenteric lymph node, and lung) from 2‐ and 24‐month‐old mice (from Envigo Ltd. Somerset, NJ, USA). The stromal cells were harvested from the subcutaneous anterior abdominal wall. Fat pads were excised and digested with 0.75 mg mL^−1^ type II collagenase (Sigma‐Aldrich) and 0.02 mg mL^−1^ DNase I at 37 °C for 50 min. Neutralized cells were centrifuged to separate mature adipocytes and the stromal‐vascular fraction. Floating adipocytes were removed, and pelleted stromal cells were passed through a 100‐μm cell strainer and stained for SA‐β‐gal activity. Lungs and lymph nodes were removed, chopped and dissociated to single‐cell suspensions by incubation for 50 min with RPMI supplemented with 1 mg mL^−1^ collagenase IV and 0.02 mg mL^−1^ DNase I at 37 °C. Intestines were removed, chopped, and dissociated to single‐cell suspensions by incubation for 50 min with RPMI supplemented with 5% FBS, 2 mm EDTA, and 0.02 mg mL^−1^ DNase I at 37 °C. Spleens were dissociated to single‐cell suspension by mechanical force. All cells were passed through 100‐μm cell strainers and stained for SA‐β‐gal activity.

### Immunoblotting

To induce p53 expression, we cultured transformed MEFs expressing tetracycline‐regulatable p53 shRNA (TRE‐shp53) in the presence of 100 ng mL^−1^ doxycycline for 5 days. Cells were lysed in RIPA buffer and equal amounts of protein were separated on 12.5% sodium dodecyl sulfate–polyacrylamide gels and transferred to polyvinylidene fluoride membranes. For detection, we used anti‐p21, anti‐p53 (both Santa‐Cruz) and anti‐β‐actin (Sigma‐Aldrich) antibodies.

### Statistical analysis

Results were compared statistically using graphpad prism software, La Jolla, CA, USA. Values were subjected to unpaired two‐tailed *t*‐tests, one‐way ANOVA followed by Tukey's honestly significant difference (HSD) post hoc test, or two‐way ANOVA. Data are presented as means ± SEM. Values of *P *<* *0.05 were considered significant.

## Funding

This work was supported by grants to V.K. from the European Research Council under the European Union's FP7 and Israel Science Foundation; and staff scientist internal grant from the Weizmann Institute of Science to Z.P. V.K. is an incumbent of the Karl and Frances Korn Career Development Chair in Life Sciences.

## Conflict of interest

None declared.

## Author contributions

A.B., P.A.K, L.Z, Y.O, L.R, and Z.P. designed, performed, and analyzed the experiments; E.V. performed the mice procedures; A.B., Z.P., and V.K. wrote the manuscript; V.K. designed and analyzed experiments and supervised the project.

## Supporting information


**Fig. S1** ImageStreamX and microscopy based analysis of senescent cells.
**Fig. S2** Autofluorescence intensities in ImageStreamX imaging channels.
**Fig. S3** Background mean pixel intensity of growing and senescent cells.
**Fig. S4** Induction of cellular senescence in tumor cells expressing tetracycline‐induced shp53.
**Fig. S5** Bright field mean pixel intensity distribution of immune and epithelial cells after SA‐β‐gal staining.
**Fig. S6** Example of gating of SA‐β‐gal positive and negative subcutaneous stromal cells.
