# Supplementary material for: Quantitative identification of senescent cells in aging and disease
Source: Aging Cell. 2017 Apr 28;16(4):661–71. doi: 10.1111/acel.12592 (PMC5506427; doi:10.1111/acel.12592)
Supplement: Supplementary file 1 — Fig. S1 ImageStreamX and microscopy based analysis of senescent cells. Fig. S2 Autofluorescence intensities in ImageStreamX imaging channels. Fig. S3 Background mean pixel intensity of growing and senescent cells. Fig. S4 Induction of cellular senescence in tumor cells expressing tetracycline‐induced shp53. Fig. S5 Bright field mean pixel intensity distribution of immune and epithelial cells after SA‐β‐gal staining. Fig. S6 Example of gating of SA‐β‐gal positive and negative subcutaneous stromal cells. [file ACEL-16-661-s001.docx]

**Supplemental Figure 1**

**
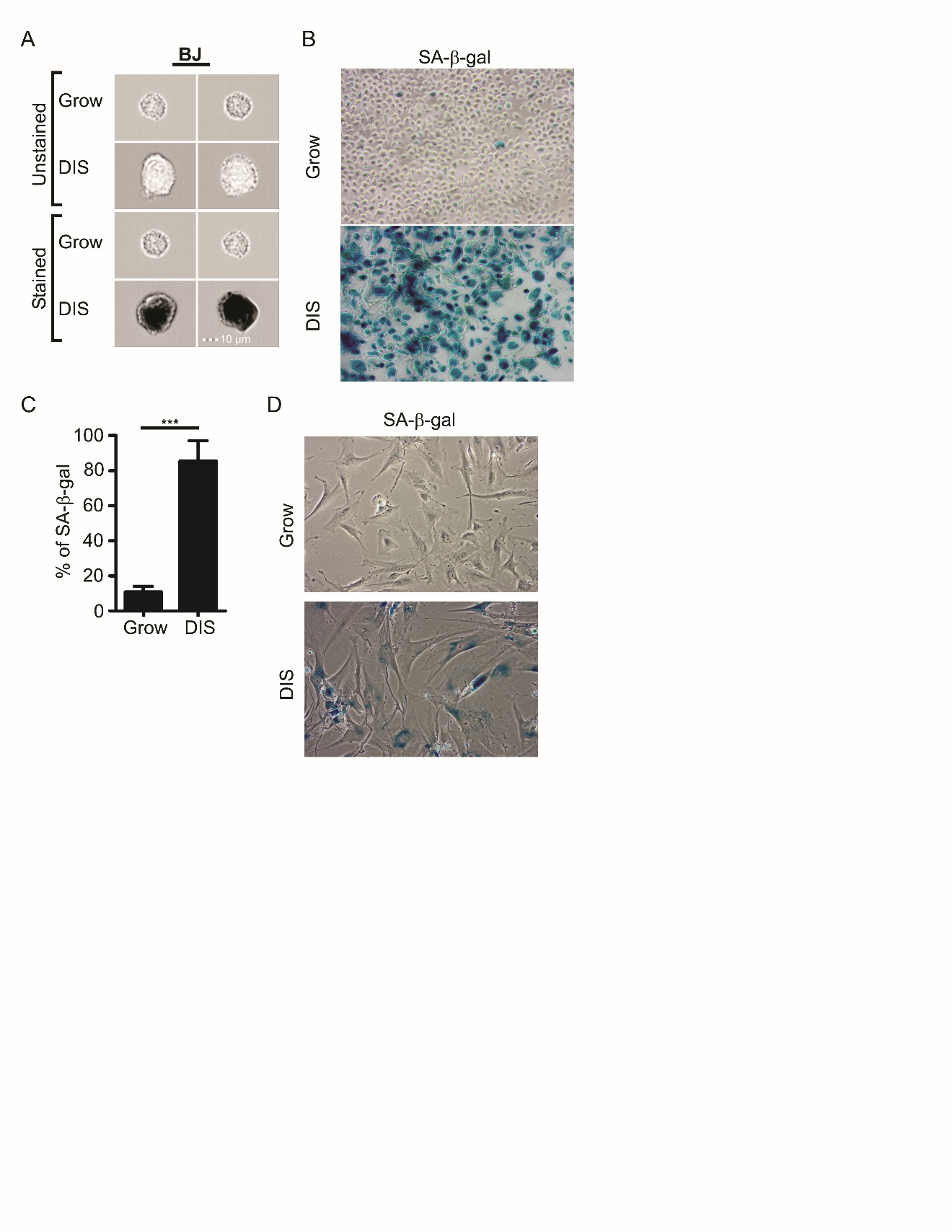
**

Supplemental Figure 1

ImageStreamX analysis of senescent cells. (A) DIS and growing BJ cells were fixed with glutaraldehyde (GA), stained for SA-β-gal or unstained, and analyzed by ImageStreamX. Representative images of the cells are shown. Bar, 10 µm. (B) DIS and growing BJ cells were fixed with PFA, stained for 8h with SA-β-gal and images were taken by microscope. Representative images are shown. (C) BJ cells were fixed with GA, stained for SA-β-gal, and positive cells were quantified by microscopy. Values are means ± s.e.m; *n* = 4, performed in 2 independent experiments; ****P* < 0.001 (Student's t-test). (D) SA-β-gal images of DIS and growing BJ cells in culture.

**Supplemental Figure 2**

**
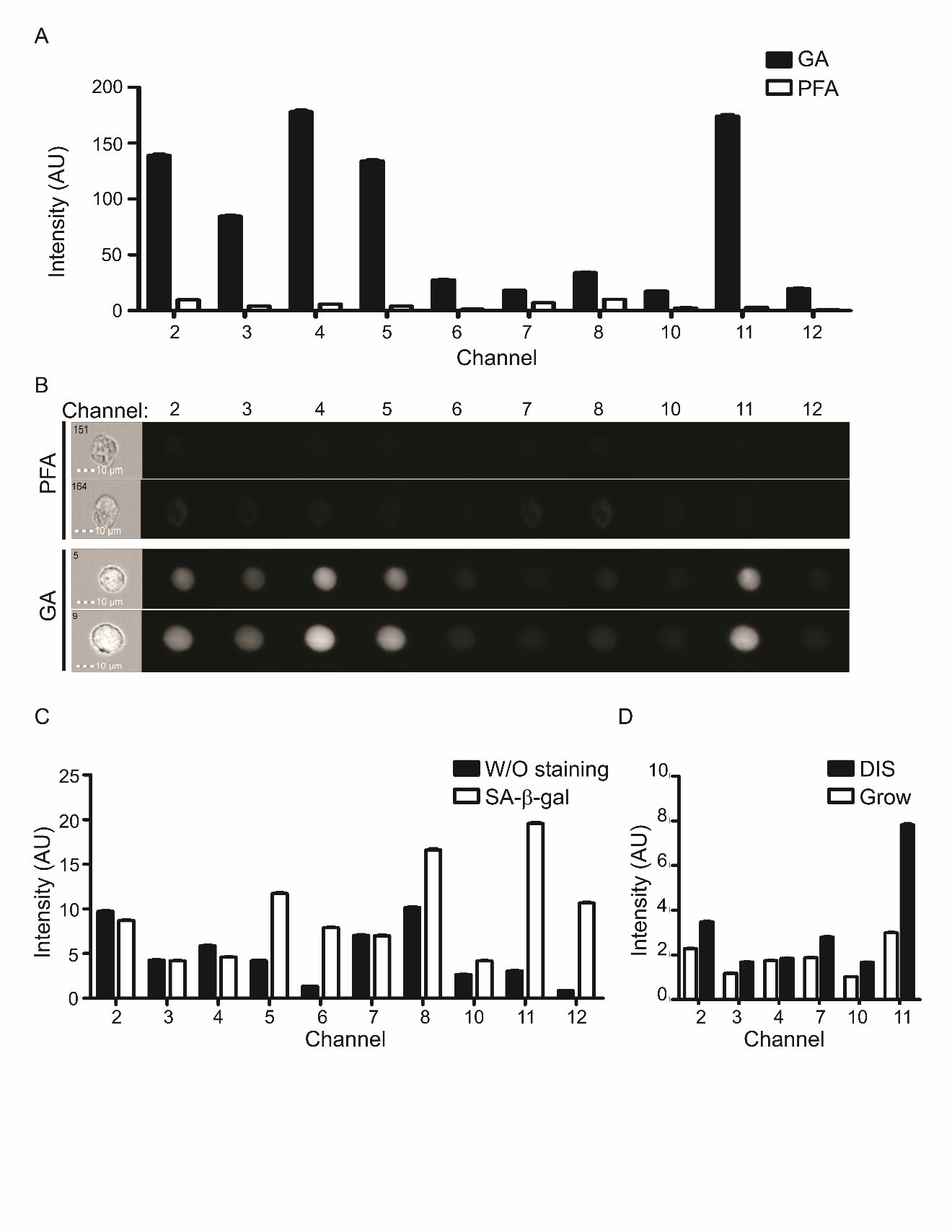
**

Supplemental Figure 2

Autofluorescence intensities in ImageStreamX imaging channels. (A) High autofluorescence in senescent mouse embryonic fibroblasts (MEFs) fixed with GA compared with those fixed with paraformaldehyde (PFA). (B) Representative images of senescent MEFs fixed with GA or PFA. (C) Quantification of autofluorescence from SA-β-gal-stained and unstained senescent MEFs fixed with PFA. (D) Comparison of autofluorescence from SA-β-gal-stained growing and DIS MEFs.

**Supplemental Figure 3**

Supplemental Figure 3

Combination of SA-β-gal with markers of senescence. (A) DIS and growing BJ cells were stained for SA-β-gal, DAPI and secondary antibody and analyzed by ImageStreamX. A representative histogram shows similar background mean pixel intensity. (B) Representative dot plot of HMGB1 and SA-β-gal staining in growing and DIS BJ cells. (C) Quantification of the overlap between SA-β-gal staining and Ki67 negative staining. Values are means ± s.e.m; *n* ≥5, performed in two independent experiments; ****P* < 0.001 (Student's t-test).

**Supplemental Figure 4**

**
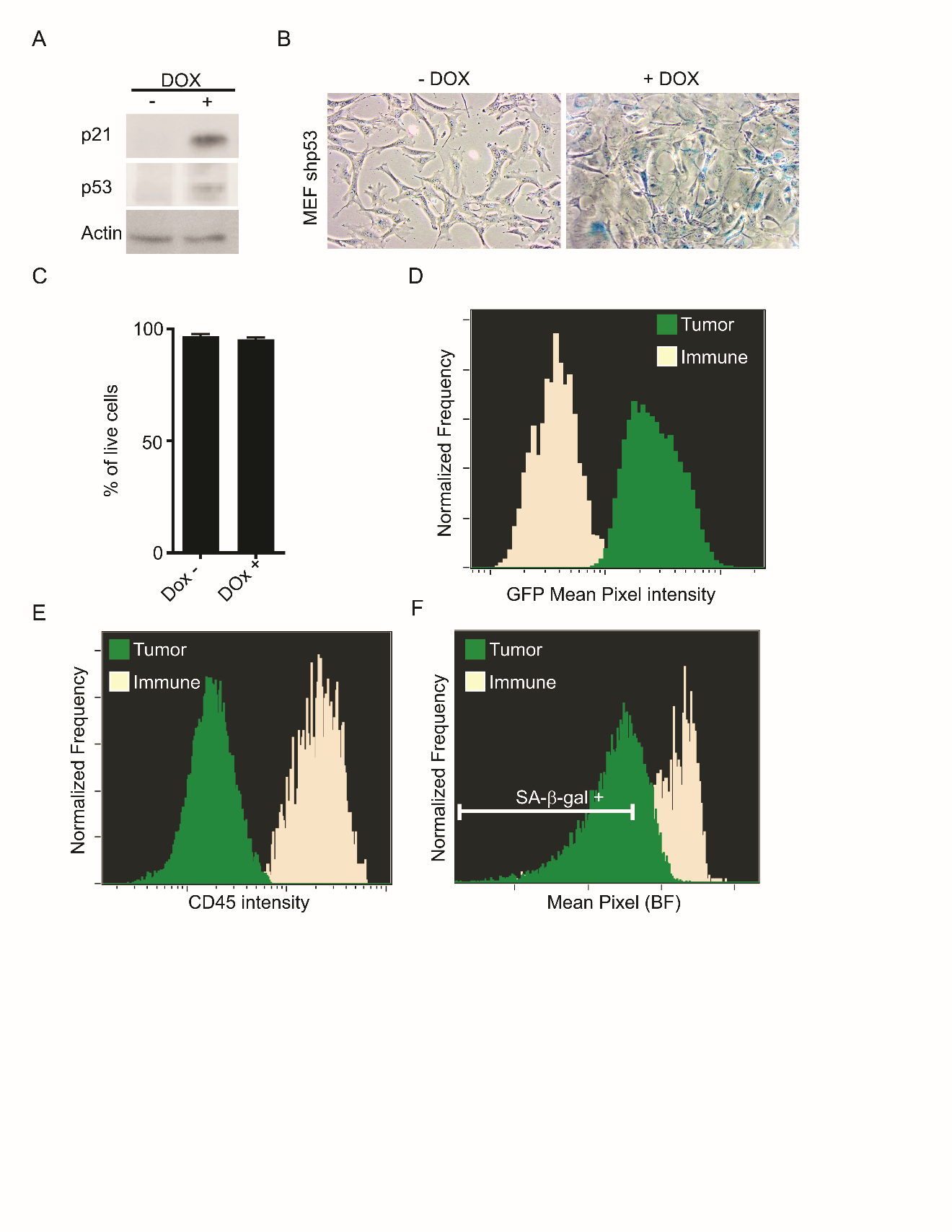
**

Supplemental Figure 4

Induction of cellular senescence in tumor cells expressing tetracycline-induced shp53. (A) Western blots of p21, p53 and actin in tumor cells upon DOX administration. (B) Microscopy images of tumor cells after treatment with DOX. (C) Cell viability following dissociation of the tumor. Values are means ± s.e.m; *n* = 4, *P* =0.27 (Student's t-test) (D) GFP mean pixel intensity distribution of immune and tumor cells extracted from tumors. (E) CD45 intensity of immune cells and tumor cells extracted from tumors. (F*)* BF mean pixel intensity of immune (CD45+) and tumor (GFP+) cells from a DOX-treated mouse.

**Supplemental Figure 5**

**
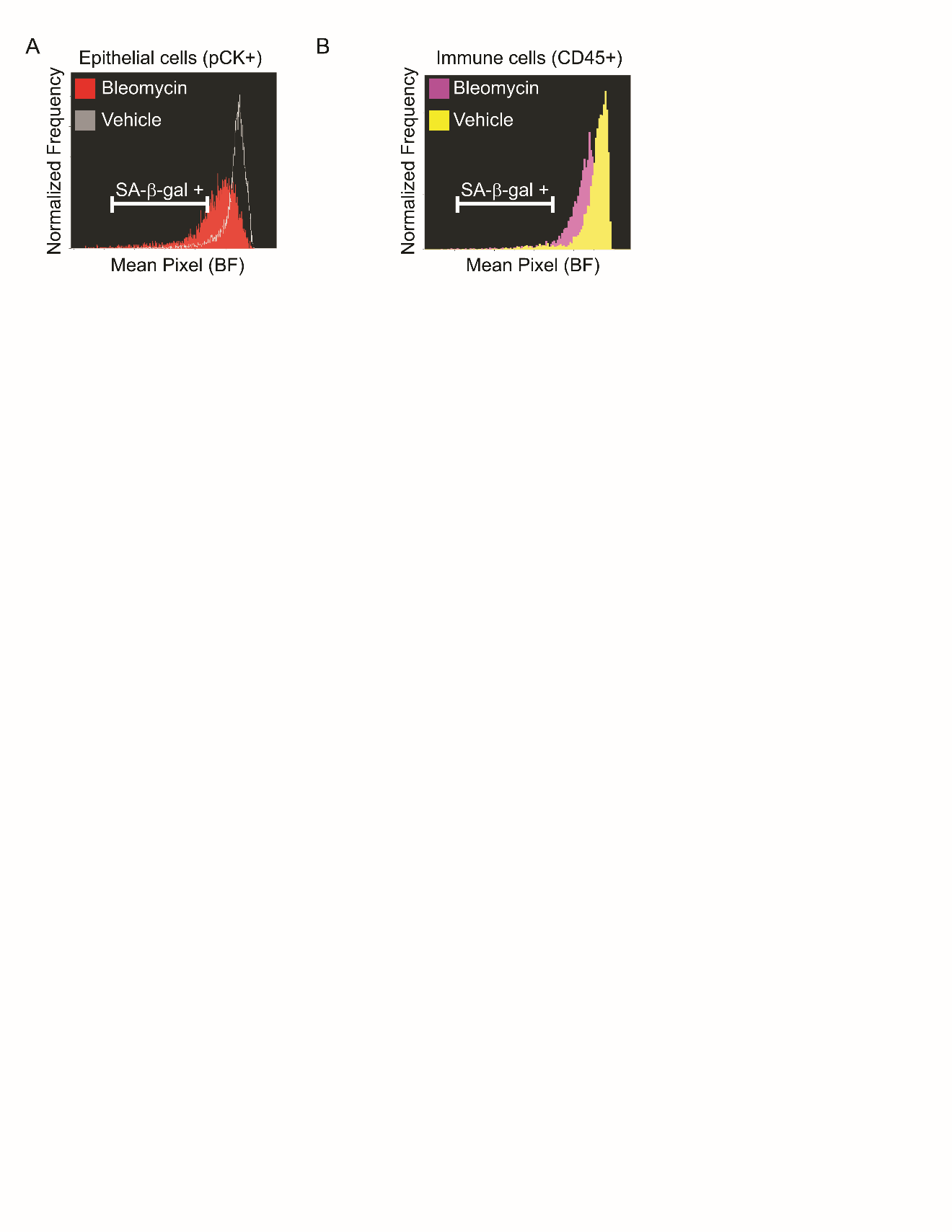
**

Supplemental Figure 5

BF mean pixel intensity distribution (A) of epithelial cells and (B) of immune cells.

**Supplemental Figure 6**


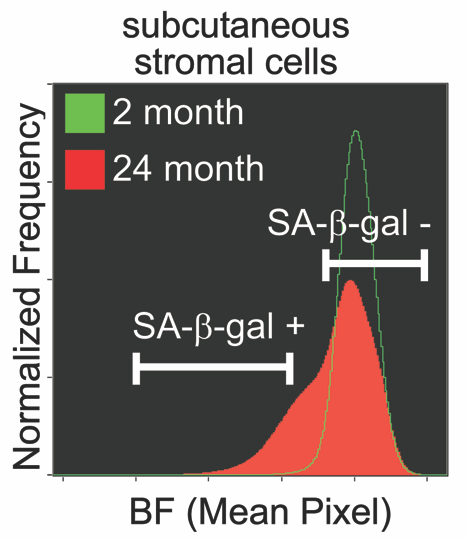


Supplemental Figure 6

Example of gating of SA-β-gal positive and negative cells.
